# Extracellular vesicle encapsulated Homer1a as novel nanotherapeutics against intracerebral hemorrhage in a mouse model

**DOI:** 10.1186/s12974-024-03088-6

**Published:** 2024-04-06

**Authors:** Xiaowei Fei, Li Wang, Ya-nan Dou, Fei Fei, Yanyu Zhang, Weihao Lv, Xin He, Xiuquan Wu, Wangshu Chao, Hongqing Chen, Jialiang Wei, Dakuan Gao, Zhou Fei

**Affiliations:** 1 Department of Neurosurgery, Xijing Hospital, Air Force Military Medical University, No. 127, Changle West Road, Xincheng District, , Shaanxi, 710032 China; 2 Department of Ophthalmology, Xijing Hospital, Air Force Military Medical University, Xi’an, 710032 Shaanxi China

**Keywords:** Intracerebral hemorrhage, Homer1a, Extracellular vehicles, A2 astrocyte, Inflammation, NF-κB

## Abstract

Homer1a and A2 astrocytes are involved in the regulation of inflammation induced by intracerebral hemorrhage (ICH). However, there is no anticipated treatment strategy based on the anti-inflammatory effect of Homer1a and A2 astrocytes. Here, we successfully induced A2 astrocytes in vitro, and then we report an efficient method to prepare Homer1a^+^ EVs derived from A2 astrocytes which making it more stable, safe, and targetable to injured neurons. Homer1a^+^ EVs promotes the conversion of A1 to A2 astrocytes in ICH mice. Homer1a^+^ EVs inhibits activation and nuclear translocation of NF-κB, thereby regulating transcription of IL-17A in neurons. Homer1a^+^ EVs inhibits the RAGE/NF-κB/IL-17 signaling pathway and the binding ability of IL-17A: IL17-AR and RAGE: DIAPH1. In addition, Homer1a^+^ EVs ameliorates the pathology, behavior, and survival rate in GFAP^Cre^Homer1^fl/−^Homer1a^±^ and Nestin^Cre^RAGE^fl/fl^ ICH mice. Our study provides a novel insight and potential for the clinical translation of Homer1a^+^ EVs in the treatment of ICH.

**Supplementary Information:**

The online version contains supplementary material available at 10.1186/s12974-024-03088-6.

## Introduction

As we know, intracerebral hemorrhage (ICH) refers to the hemorrhage caused by the rupture of blood vessels in the brain parenchyma due to hypertension or trauma et al., accounting for 20–30% of the total stroke, [1] and the mortality rate in the acute phase is 30–40% [2, 3]. ICH causes inflammation in the brain and accelerates brain edema, resulting in neuronal damage, and lead to elevated disability and mortality of patients [4]. However, there is currently no definitive therapeutic countermeasures to deal with the inflammatory response induced by established ICH.

Homer scaffold protein 1 (Homer1) is widely distributed in the central nervous system (CNS) and is mainly divided into long Homer1b/c protein and short Homer1a protein [5]. Due to the lack of CC domain, [6] Homer1a can only bind Homer1-related ligands through EVH1 domain, thereby rearranging molecules on the neuronal membrane and playing a negative role in regulating Homer1b/c [7, 8]. Our research team has focused on the study of Homer1a protein and found that Homer1a is involved in protecting PC12 cells from oxygen free radical damage [9]. In ischemia–reperfusion injury of retinal ganglion cells, up-regulation of Homer1a expression inhibits phosphorylation of the Erk pathway, thereby reducing ganglion cell apoptosis [10]. In the oxidative stress injury of HT-22 cells induced by glutamate, Homer1a alleviated the intracellular calcium overload of HT-22 cells by inhibiting the manipulation of calcium influx in the calcium pool, thus may play a fundamental role in neuroprotection [11]. The anti-inflammatory and anti-oxidative stress effects of Homer1a suggest that Homer1a may be a potential therapy to address current clinical challenges in ICH treatment. However, Homer1a protein is synthesized by the immediate-early gene, and the stability and expression of Homer1a are very poor under physiological conditions [12]. Presently the critical issue is how to improve the stability of Homer1a and selectively target ICH tissues.

Extracellular vehicles (EVs) are a class of nanoscale extracellular vesicular substances secreted by cells with phospholipid bilayer structures [13, 14]. EVs are important carriers for cell-to-cell signal transmission [14]. It has the function of transmitting bioactive molecules between cells to regulate target cells. In neurological diseases, EVs have been reported to participate in neural repair in a variety of ways [15]. For example, the function of neuron-derived small EVs might be regulated by the status of neurotransmitter balance and that EVs might affect amyloid βtoxicity on neurons [16]. The unique advantage of EVs over existing delivery systems is their natural origin, which allows them to avoid phagocytosis, prolong the half-life of therapeutic agents, and reduce immunogenicity [17]. Therefore, EV-based drug delivery systems may be an attractive candidate to manipulate Homer1a for effective treatment of ICH.

Here, we report an efficient method to prepare Homer1a-loaded EV (Homer1a^+^ EV) and investigate the therapeutic effect of Homer1a^+^ EV in mouse models of ICH in vivo and in neuronal ICH models in vitro. It was found that Homer1a^+^ EV enhanced the stability of Homer1a protein, and orthotopic injection of EVs effectively targeted damaged neurons. In addition, Homer1a^+^ EV also promoted the transformation of astrocyte phenotype from A1 to A2 around ICH, thereby significantly improving the pathological damage caused by ICH. Specifically, we found that Homer1a^+^ EV inhibited the RAGE/NF-κB/IL-17 signaling pathway in neurons, attenuated nuclear translocation of NF-κB, and inhibited the binding ability of IL-17A: IL17-AR and RAGE: DIAPH1. Our results strongly support the use of A2 astrocyte derived EVs as a multifunctional delivery system for Homer1a protein and the use of Homer1a^+^ EVs for the treatment of acute ICH.

## Materials and methods

### Animals

All animal experiments were performed in accordance with protocols approved by the Institutional Ethics Committee of Xijing Hospital. All experimental procedures were approved by the Institutional Animal Care and Use Committee of Air Force Military Medical University. Male C57BL/6 J and Nestin^Cre^RAGE^fl/fl^ mice were purchased from Cyagen Biotechnology Co., Ltd (Jiangsu, China). Male Homer1a conditional knockout mice (GFAP^Cre^Homer1^fl/−^Homer1a^±^) were designed and purchased from Shanghai Model Organisms Center, Inc. All mice were maintained in the same environment.

### Extraction and culture of primary cortical neurons and astrocytes

Neonatal C57BL/6 J mice were used to extract primary neurons and astrocytes using a stereomicroscope. The culture dish was coated with 0.2 mg/mL of poly-L-lysine (Sigma-Aldrich) overnight at 37 ℃, washed three times with sterile water and placed in an incubator for use. The brain tissue was minced with sterile ophthalmic scissors, digested with 0.25% trypsin for 5 min at 37 °C before the brain tissue was centrifuged at 1000 rpm for 5 min.

For the extraction of primary neurons, the complete Dulbecco’s modified Eagle’s medium (DMEM, Gibco) was used for appropriate dilution and the cell suspension was made into a seed plate. After 4–6 h, DMEM was replaced with Neurobasal medium (Gibco) for primary neurons which containing 0.25% glutamine (Sigma-Aldrich), 1% penicillin/streptomycin (Gibco) and 2% B-27 supplement (Gibco). Neurons monolayers were obtained at the bottom of the dish after 7 days. The neurons were identified by morphological analysis and neuron-specific enolase (NSE) staining.

For the extraction of primary astrocytes, the digested tissue was cultured in F-12 medium enriched with 10% fetal bovine serum (FBS, Gibco), 0.224% NaHCO_2_ (Sigma-Aldrich), and 1% penicillin/streptomycin at 37 °C in the presence of 5% CO_2_. Astrocyte monolayers were obtained at the bottom of the dish after 2 weeks. The astrocytes were identified by morphological analysis and glial fibrillary acidic protein (GFAP) staining. Before further treatment, the medium containing 0.5% FBS was replaced to ensure that the cells were in a resting state.

### Induction of astrocytes with A1 and A2 phenotypes

A1 astrocytes were generated as described by Liddelow et al. [18]. Astrocyte conditional medium (ACM) was prepared in DMEM supplemented with C1q (400 ng/mL, Novus protein, NBP2-62410), TNF-α (30 ng/mL, CST, 8902) and IL-1α (3 ng/mL, Peprotech, 400-01). Conversion of astrocytes from A1 to A2 was examined by protein and mRNA expression of C3 and S100A10 after the cells were cultured in ACM for 24 h.

Primary neurons (1 × 10^5^/200 μL) were cultured in the upper transwell compartment (HTS Transwell-24 units w/0.4 μm pore polycarbonate membrane and 6.5 mm inserts, Sigma-Aldrich) and primary astrocytes (2 × 10^5^/600 μL) were cultured in the lower transwell compartment. Cells were stably adherent after 24 h of culture in a common oxygenated incubator and were placed in a hypoxic incubator for another 12 h after changing glucose-free DMEM medium. Astrocytes were collected from the lower compartment of the transwell and changes in the expression of cell markers C3 and S100A10 were detected by flow cytometry.

### Preparation of Homer1a^+^ EVs

To overexpress Homer1a in donor A2 astrocytes, primary astrocytes in a resting state were transfected with Homer1a^+^ pWPI plasmid (Hanbio, Shanghai) using jetPRIME in vitro DNA transfection reagent (Polyplus). After 8 h of transfection, the modified astrocytes were induced into Homer1a^+^ A2 astrocytes according to the methods mentioned above. Then, the medium was replaced with Serum-free EVs culture Media (Umibio) and Homer1a^+^ A2 astrocytes were cultured in ordinary oxygen-containing incubator for 48 h. The supernatants were subsequently subjected to sequential centrifugation steps. Briefly, the supernatant was centrifuged for 10 min at 1500 *g* to eliminate precipitation. Then, the supernatant was centrifuged for 30 min at 10,000 *g*, 4 ℃ to remove small precipitation, and then the supernatant was sucked into a new centrifuge tube. Supernatants were further concentrated for 1 h at 100,000 *g* (Beckman, Optima XE-100) after filtered through a 0.22 μm filter. EVs were resuspended with 10–15 mL PBS and centrifuged at 100,000 *g* for 1 h to pellet the EVs. The pellet was washed one time and resuspended in sterile PBS or culture medium for the following experiments. The transcript sequence for overexpressing Homer1a was NM_011982.

### Nanoparticle tracking analysis (NTA)

The ZetaView sample tank was cleaned with pure water for 3 times. EVs were diluted to appropriate concentration with PBS and injected into ZetaView (particle metrix) sample pool through syringe for detection. Briefly, 2 μL resuspended EVs were sucked into 2 mL PBS and mixed well. Then, the diluted sample was added into the sample pool, the real-time dynamic image of EVs particles were observed through the computer display screen, and the concentration distribution of EVs were detected.

### Transmission electron microscopy (TEM)

Twenty μL of the resuspended EVs were added dropwise to 200-mesh grids and incubated at room temperature for 10 min, then the grids were negatively stained with 2% phosphotungstic acid for 3 min, and the remaining liquid was removed by filter paper. Then observed with a HT7800 transmission electron microscope.

### Labeling of Homer1a^+^ EVs

To obtain DID-labeled or PKH67-labeled Homer1a^+^ EVs, the donor A2 astrocytes cells were labeled with PKH67 (Sigma-Aldrich) or DID (Invitrogen) according to the manufacturer’s protocol, respectively. Then, cells were washed twice with 0.5% FBS to remove excess dye. After the cells were treated differently according to the experimental design and grouping, the supernatants were collected and Homer1a^+^ EVs were isolated as described above. In this way, we can remove the free dye in pellet to the greatest extent.

### Animal models and therapeutic experiments

Establishment of ICH model was performed as previously described [19, 20]. Briefly, the mice were anesthetized with 4% chloral hydrate (400 mg/kg) injected intraperitoneally. The needle was inserted into the brain through the burr hole with a microinjection pump (0.2 mm anterior and 2.5 mm lateral to the bregma depth, 3.5 mm from the bone surface). Thirty mL of autologous blood obtained from the femoral artery were injected within 10 min. To avoid postsurgical dehydration, normal saline (0.5 mL) was subcutaneously injected into each mouse immediately after the surgery.

In therapeutic experiments, EVs were treated in situ immediately after ICH model construction. The therapeutic dose is 100ug or 200 μg/10 μL PBS for each mouse according to the experimental design. At the same time points on the first and second days after operation, mice were treated with EVs in situ under isoflurane anesthesia, with the same dose as on the first day. On the third day after operation, the mice that had undergone three treatments were euthanized under anesthesia, and the brain tissue was used in the follow-up experiment. In rescue experiment, the therapeutic dose of Dehydroxymethylepoxyquinomicin (DHMEQ, MCE; USA) is 4 mg/kg/10 μL for three consecutive days.

### Cell models and therapeutic experiments

The primary astrocytes were cultured in ACM medium for 24 h and then induced into A1 astrocytes. The successfully induced A1 astrocytes were divided into three groups: ACM group, EVs-100 μg group and EVs-200 μg group.

We used erythrocyte lysis stimulation (ELS) to induce mouse primary neurons as the inflammation model [21]. The whole blood of mice and Red Blood Cell Lysis Buffer (Solarbio, Beijing) were mixed in a volume ratio of 1:3 to obtain red blood cell lysate. The primary neurons were cultured in the medium containing 5% red blood cell lysate for 24 h and then served as the ICH model group in vitro. The successfully induced ICH model neurons were divided into three groups: ICH group, EVs-100 μg group and EVs-200 μg group.

All cells were cultured with 15 mL medium in a 75 cm culture flask for 48 h after EVs or DHMEQ (5 μg/mL) treatment.

### Experimental grouping

Sixty C57BL/6 J mice were divided into Sham group (n = 10) and model group (n = 50). The model group was divided into ICH group (n = 10), 100 μg group (n = 10), 200 μg group (n = 10), DHMEQ group (n = 10) and DHMEQ + EVs 200 μg group (n = 10) according to different treatment was given.

Eighty GFAP^Cre^Homer1^fl/−^Homer1a^±^ mice or Nestin^Cre^RAGE^fl/fl^ mice were divided into Sham group (n = 20) and model group (n = 60). The model group was divided into ICH group (n = 20), 100 ug group (n = 20) and 200 ug group (n = 20) according to whether EVs treatment was given. Ten mice in each group were used for the calculation of survival analysis. The behavioral score of each mouse was evaluated before euthanasia to ensure that the sample size of behavioral data was 10 per group.

The fresh brain tissues of 5 mice in each group were used for molecular biology experiments or extraction of primary cells. The brain tissues of the remaining 5 mice in each group were fixed with paraformaldehyde for pathological section detection experiments. In addition, the number of mice was at least 3 per group in all experiments under normal mortality.

Twenty transgenic NF-κB-Luciferase reporter gene mice were divided into Sham group (n = 5) and model group (n = 15). The model group was divided into ICH group (n = 5), 100 ug group (n = 5) and 200 ug group (n = 5) according to whether EVs treatment was given.

### Data processing

For molecular biology experiments, three technical replicate experiments were performed for each mouse and the data were averaged. The three mean values (n = 3/group) obtained from three mice in each group were used for statistical comparisons between groups. In vitro experiments, assays were performed on cells in three wells for each experiment to obtain an average count, and in three independent biological replicates.

For pathological experiments, one section from each of three mice in each group was observed under immunofluorescence laser confocal microscopy, and three fields randomly selected from each section were used to quantify the detection indicators and the data were averaged. The three mean values (n = 3/group) obtained for each group were used for statistical comparisons between groups.

### Statistical analysis

Prism 8 for macOS software was used for the statistical analyses. Power analysis is used to ensure proper sample size. All values for each group are presented as mean ± SD. Parametric and nonparametric tests were used according to the homogeneity of variance. According to different comparison situations, statistical differences were analyzed using Student’s t-test or one-way ANOVA, as appropriate, with Sidak’s or Turkey’s multiple comparisons test. *P* < 0.05 indicated that the difference was statistically significant.

Identification of transgenic mice, Hematoxylin–eosin (HE) staining, Nissl staining, TUNEL assay, Immunohistochemistry, Western blot (WB), Quantitative polymerase chain reaction (qPCR), Longa scores, Co-immunoprecipitation (Co-IP), Enzyme linked immunosorbent assay (ELISA), Flow cytometry, Double luciferase reporter assay, Shotgun experiments and Protein chip assay.

All experiments above are describe detailed in Additional file 1: Materials and methods.

## Results

### Preparation and characteristics of Homer1a^+^ EVs derived from A2 astrocytes

**Fig. 1:**
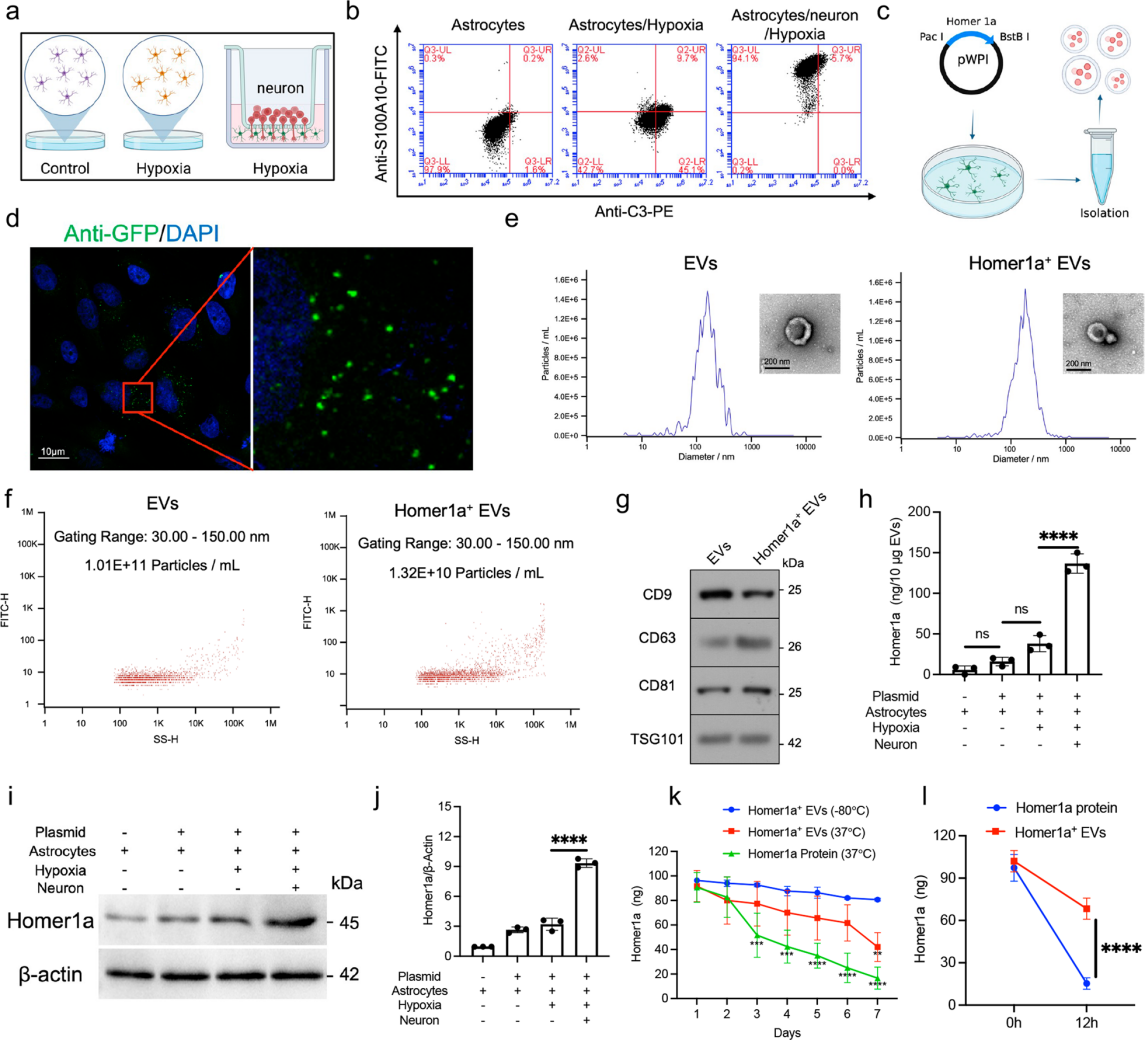
Preparation and characteristics of Homer1a^+^ EVs. **a** Schematic diagram of A2 astrocyte induction. **b** Flow cytometry detection of the marker S100A10/C3 of A1/A2 astrocytes. **c** Schematic diagram of Homer1a^+^ EVs extraction.** d** Fluorescence of A2 astrocytes transfected with Homer1a-pWPI plasmid. **e** Size distribution and representative TEM images of EVs and Homer1a^+^ EVs. **f** The concentration of EVs and Homer1a^+^ EVs measured by nanoflow.** g** Western blotting analysis of EV-associated markers (CD9, CD63, CD81 and TSG101) in EVs and Homer1a^+^ EVs.** h** ELISA analysis of Homer1a in EVs from different treated cells [F (3, 8) = 147.7, P < 0.0001]. **i** WB analysis of Homer1a in EVs from different treated cells. **j** Quantification of result in panel i [F (3, 8) = 250.8, P < 0.0001]. **k** Homer1a concentration was detected by ELISA analysis at different time points under different conditions, including put in − 80 °C or 37° for 7 days. [F_interaction_(12, 42) = 2.784, P = 0.0071].** l** Comparison of the stability of Homer1a^+^ EVs and free Homer1a protein in pH 5.5 solution for 12 h. [F_interaction_(1, 8) = 32.00, P = 0.0005]. The data were analyzed using one-way (**h**, **j**) or two-way (**k**, **l**) analysis of variance and all data are expressed as the mean ± standard deviation. ***P < 0.001 and ****P < 0.0001 represents a statistically significant difference between the two groups. ns: no statistical difference. The blots are representative of other replicates in those groups

Substances supporting the growth, health and survival of neurons are secreted by A2 astrocytes near the cerebral ischemia site [22, 23]. We constructed the microenvironment of A2 astrocytes in vitro (Fig. 1a). Flow cytometry showed that hypoxia would lead to the simultaneous transformation of astrocyte phenotype to A1 and A2. However, co-culture with neurons in hypoxic environment induced the conversion of A1 astrocytes to A2, which indicates that we have successfully induced A2 astrocytes in vitro (Fig. 1b). In addition, Homer1a-pWPI plasmid was constructed and transfected into successfully induced A2 astrocytes (Fig. 1c). We found that scattered punctate green fluorescence appeared both intracellularly and extracellularly (Fig. 1d), therefore, we speculated that the overexpressed Homer1a might be wrapped in a certain vesicle structure. Serum-free EVs culture media was used to culture cells and then the supernatant was collected to extract EVs by high-speed centrifugation. The diameter of extracted EVs detected by NTA was approximately 50–500 nm with a mean diameter of 120 nm (Fig. 1e). The morphology detected by TEM appeared heterogeneous with a mixture of both small exosome-like and large micro-vesicle-like particles (Fig. 1e). The concentration of EVs detected by nanoflow was approximately 10^10 particles per milliliter (Fig. 1f). In addition, results of WB showed that EVs and Homer1a^+^EVs were positive for the expression of EVs markers CD9, CD63, CD81 and TSG101 (Fig. 1g). These results indicate that we have extracted EVs from A2 astrocytes successfully.

To examine the expression levels of Homer1a in EVs extracted from A2 astrocytes transfected with the Homer1a-pWPI plasmid, Elisa (Fig. 1h), WB and qPCR were used to detect the protein and mRNA levels of Homer1a in EVs extracted from different groups. The protein (Fig. 1i) and mRNA (Fig. 1j) of Homer1a are expressed at a higher level in EVs extracted from A2 astrocytes transfected with the Homer1a-pWPI plasmid (136.7 ± 11.93 ng/10 ug, Fig. 1h). In addition, compared with Homer1a protein, Homer1a^+^ EVs degrade more slowly at 37 ℃. Homer1a protein level of Homer1a^+^EVs still maintain 80.67 ± 1.15% of the original after 7 days at – 80 ℃ (Fig. 1k). It is reported that nucleic acids and proteins show high stability inside EVs. We found that Homer1a protein was well protected in the EVs suspended in pH 5.5 solution for 12 h (Fig. 1l). Given that the transience and instability of Homer1a has greatly hindered its clinical use, our approach designed to deliver Homer1a using EV could overcome critical translational barriers.

### Neurons targeting of Homer1a^+^ EVs

We administered 100 μg Homer1a^+^ EVs daily for 3 consecutive days to ICH mice (Additional file 1: Fig. S1a). The result of IF showed that DID-labeled EVs mainly co-localized with neurons (Additional file 1: Fig. S1b). DID and NeuN double-positive cells accounted for 10.79 ± 1.52% (Additional file 1: Fig. S1c). To further validate that Homer1a^+^ EVs target neurons. Primary neurons from mice were extracted and treated with 100 μg Homer1a^+^ EVs (Additional file 1: Fig. S1d). We used ELS to induce mouse primary neurons as the inflammation model. Compared with the control group, neurons treated with ELS highly expressed TNF-α, indicating that the neuronal in vitro ICH model was successfully induced. Following treatment of cells with PKH-67-labeled Homer1a^+^ EVs (100 μg), we found that neurons in the ICH group expressed intense PKH-67 fluorescence compared to the control group (Additional file 1: Fig. S1e). In addition, the flow cytometry results also suggested a higher proportion of neurons co-expressing NeuN-PE and PHK67-FITC in the ICH group compared with the control group (Additional file 1: Fig. S1f). These results indicate that Homer1a^+^ EVs may mainly target neurons after ICH in mice, rather than microglia and astrocytes.

### Homer1a+ EVs protect against the pathology, behavior, and survival rate in ICH GFAP^Cre^Homer1^fl/−^Homer1a^±^ mice

**Fig. 2:**
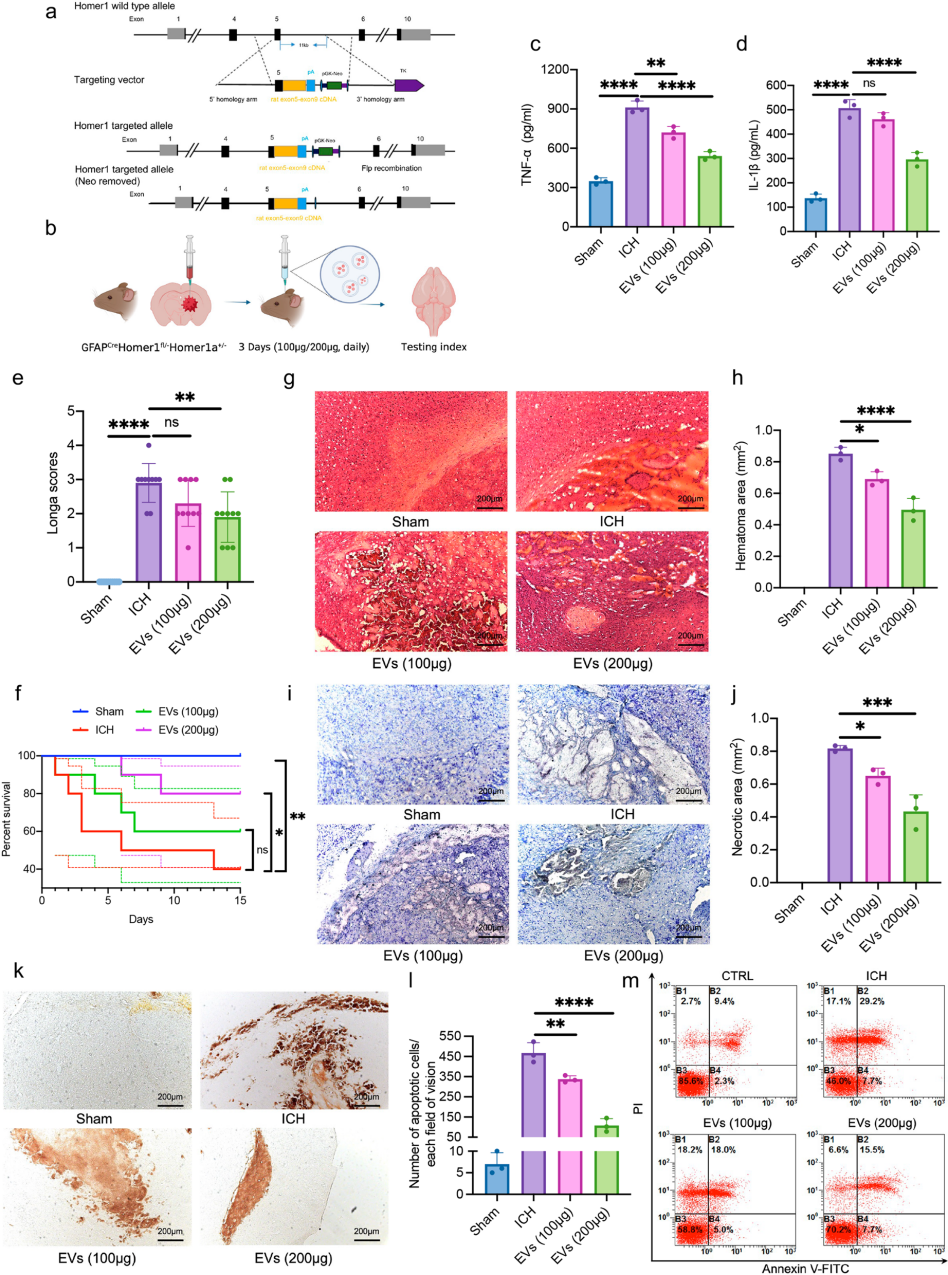
Homer1a^+^ EVs has therapeutic effect on ICH GFAP^Cre^Homer1^fl/−^Homer1a^±^ mice. **a** Schematic diagram of Homer1a conditional knockout Mouse model (C57BL/6 J) by CRISPR/Cas-mediated genome engineering.** b** Schematic diagram of GFAP^Cre^Homer1^fl/−^Homer1a^±^ mice injected with Homer1a^+^ EVs.** c** Detection of TNF-α level in brain tissue of each group by ELSIA [F (3, 8) = 111.3, P < 0.0001].** d** Detection of IL-1β level in brain tissue of each group by ELSIA [F (3, 8) = 111.1, P < 0.0001]. **e** Longa scores of mice in different groups [F (3, 36) = 47.47, P < 0.0001].** f** Survival curve of mice in each group (n = 10/group) [log-rank (Mantel–Cox) test: Chi-square = 10.40; df = 3; P = 0.0154]. **g** Representative photographs of HE staining of brain tissue in each group.** h** Quantification of result in panel g [F (3, 8) = 179.3, P < 0.0001].** i** Representative photographs of Nissl staining of brain tissue in each group.** j** Quantification of result in panel i [F (3, 8) = 118.2, P < 0.0001].** k** Representative photographs of TUNEL staining of brain tissue in each group.** l** Quantification of result in panel k [F (3, 8) = 131.0, P < 0.0001]. **m** Representative photographs of flow cytometry of primary neuron in each group. The data were analyzed using one-way analysis of variance and all data are expressed as the mean ± standard deviation. *P < 0.05, **P < 0.01 ***P < 0.001 and ****P < 0.0001 represents a statistically significant difference between the two groups. ns: no statistical difference

To explore the therapeutic effect of Homer1a^+^ EV on ICH mice, we used genetic engineering technology (Fig. 2a) to construct transgenic mice with astrocyte conditionally knockout Homer1a (GFAP^Cre^Homer1^fl/−^Homer1a^±^). Knockout of *Homer1a* gene in mouse astrocytes effectively eliminated the interference of endogenous Homer1a^+^ EV to the experimental results. ICH mice were treated with Homer1a^+^ EVs at different doses of 100 μg or 200 μg for 3 consecutive days after operation (Fig. 2b). We found that compared with ICH group, Homer1a^+^ EVs treatment significantly reduced the expression of inflammatory factors TNF-α (Fig. 2c) and IL-1β (Fig. 2d) in brain tissue around ICH hemorrhage. Results of behavior (Fig. 2e) and survival analysis (Fig. 2f) showed that there was no statistical difference between the 100 μg dose of Homer1a^+^ EV group and ICH group, but the 200 μg dose of Homer1a^+^ EVs treatment significantly decreased the Longa score and mortality (Log-rank Mantel-Cox test, Chi square: 10.40; df: 3; *P* = 0.0154) of ICH mice. Pathological examination of brain tissue, including HE (Fig. 2g, h), Nissl (Fig. 2i, j) and TUNEL (Fig. 2k, l) staining, revealed that Homer1a^+^ EVs treatment after ICH decreased the area of hemorrhage and necrosis in brain tissue, improve the morphology of Nissl bodies in neurons, and reduce the number of apoptotic cells in bleeding tissue. In addition, the primary neurons were extracted from the brain tissues around the hemorrhage of mice in each group and apoptosis levels of primary neurons were detected by flow cytometry. The results indicated that the number of primary neuron apoptosis in Homer1a^+^ EVs treated group was less than that in ICH group, and the difference was statistically significant (Fig. 2m). In addition, it is important that the therapeutic effect of Homer1a^+^ EV was positively correlated with the dose. These results indicate that Homer1a^+^ EVs protect against the pathology, behavior, and survival rate in ICH GFAP^Cre^Homer1^fl/−^Homer1a^±^ mice.

### Homer1a^+^ EVs promotes the conversion of A1 astrocytes to A2 astrocytes in ICH mice

**Fig. 3:**
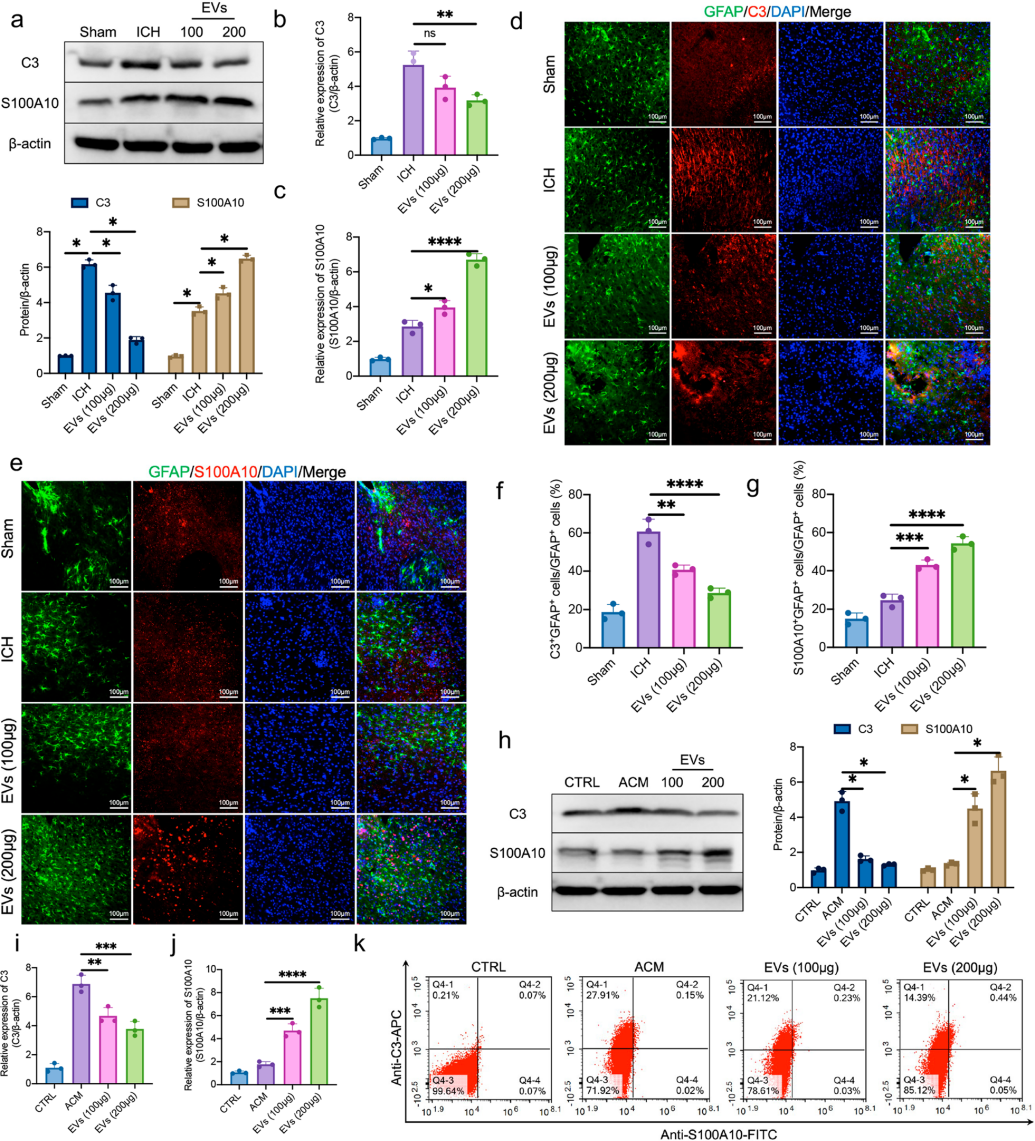
Transcription and translation levels of C3 and S100A10 in primary astrocytes and mice ICH model after Homer1a^+^ EVs treatment. **a** Protein expression and quantification of C3 and S100A10 in brain tissues after Homer1a^+^ EVs treatment [C3: F (3, 8) = 249.8, P < 0.0001; S100A10: F (3, 8) = 311.3, P < 0.0001]. **b** mRNA level of C3 [F (3, 8) = 32.8, P < 0.0001)].** c** mRNA level of S100A10 [F (3, 8) = 170.4, P < 0.0001)].** d** Representative photographs of C3 and GFAP co-staining of frozen sections of brain tissue in different group.** e** Representative photographs of S100A10 and GFAP co-staining of frozen sections of brain tissue in different group.** f** Quantification of result in panel d [F (3, 8) = 54.9, P < 0.0001)]. **g** Quantification of result in panel e [F (3, 8) = 97.48, P < 0.0001)].** h** Protein expression and quantification of C3 and S100A10 in primary neurons ICH models after Homer1a^+^ EVs treatment [C3: F (3, 8) = 113.1, P < 0.0001; S100A10: F (3, 8) = 63.67, P < 0.0001].** i** mRNA level of C3 [F (3, 8) = 65.35, P < 0.0001)].** j** mRNA level of S100A10 [F (3, 8) = 90.73, P < 0.0001)].** k** Representative photographs of flow cytometry of primary astrocyte in each group. The data were analyzed using one-way analysis of variance and all data are expressed as the mean ± standard deviation. *P < 0.05, **P < 0.01 ***P < 0.001 and ****P < 0.0001 represents a statistically significant difference between the two groups. ns: no statistical difference. The blots are representative of other replicates in those groups

To investigate whether Homer1a^+^ EVs derived from A2 astrocytes can promote the conversion of astrocytes phenotype after ICH, we detected the transcription and translation levels of A1 marker C3 and A2 marker S100A10 in peri-hemorrhagic tissues. It is found that Homer1a^+^ EVs treatment significantly decreased expression of C3 and increased expression of S100A10 after ICH. The difference of protein expression between the treatment group and ICH group was statistically significant (Fig. 3a). The results of qPCR revealed that there was no significant difference in mRNA level of C3 between 100 μg EVs group and ICH group, and the mRNA level of C3 in 200 μg EVs group was significantly lower than that in ICH group (Fig. 3b). Both 100 μg and 200 μg EVs treatment significantly ascended mRNA level of S100A10 after ICH (Fig. 3c). In addition, the results of IF indicated that the number of C3^+^GFAP^+^ cells in Homer1a^+^ EVs treatment group was lower than that in ICH group (Fig. 3d, f), while the number of S100A10^+^GFAP^+^ cells was higher (Fig. 3e, g). Furthermore, primary astrocytes were extracted and cultured in ACM medium to be induced into A1 phenotype. After Homer1a^+^ EVs treatment, the expression of C3 protein in A1 type astrocytes declined and S100A10 protein raised (Fig. 3h). The changes of C3 and S100A10 protein expression were positively correlated with the dose of Homer1a^+^ EVs. The same results were obtained for mRNA level detection (Fig. 3i, j). Flow cytometry also showed that A1 astrocytes transformed into A2 phenotype after Homer1a^+^ EVs treatment (Fig. 3k). These data suggested that Homer1a^+^ EVs promotes the conversion of A1 astrocytes to A2 astrocytes in ICH mice.

### Homer1a^+^ EVs inhibit RAGE/NF-κB/IL-17 signaling pathway after ICH

**Fig. 4:**
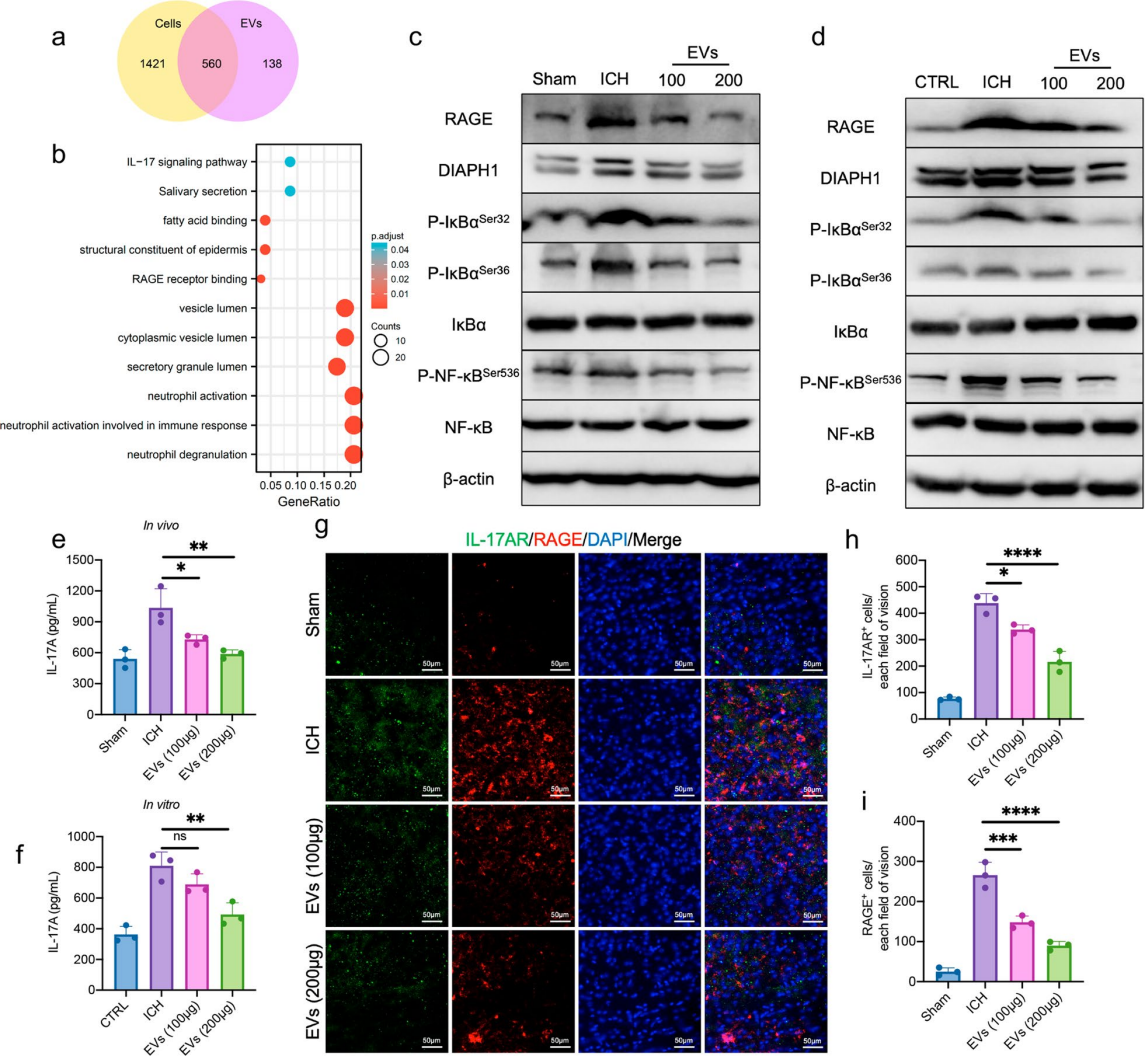
RAGE/NF-κB/IL-17 signaling pathway was regulated by Homer1a^+^ EVs in vivo and in vitro. **a** Differential expression protein between cells and EVs. **b** KEGG analysis of 138 proteins detected only in EVs. **c** The expression level of RAGE/NF-κB/IL-17 signaling pathway related proteins was detected by WB in vivo. **d** The expression level of RAGE/NF-κB/IL-17 signaling pathway related proteins was detected by WB in vitro. **e** Detection of IL-17A level in vivo by ELSIA [F (3, 8) = 13.02, P = 0.0019]. **f** Detection of IL-17A level in vitro by ELSIA [F (3, 8) = 22.30, P = 0.0003]. **g** Representative photographs of IL-17AR and RAGE co-staining of frozen sections of brain tissue in different group. **h** Quantification of IL-17AR^+^ cells in panel **g** [F (3, 8) = 91.74, P < 0.0001)]. **i** Quantification of RAGE^+^ cells in panel g [F (3, 8) = 84.96, P < 0.0001)]. The data were analyzed using one-way analysis of variance and all data are expressed as the mean ± standard deviation. *P < 0.05, **P < 0.01 ***P < 0.001 and ****P < 0.0001 represents a statistically significant difference between the two groups. ns: no statistical difference. The blots are representative of other replicates in those groups

Shotgun technology was performed to assess the protein composition of Homer1a^+^ EVs with their parent neuron cells as the control. It was found that 1421 proteins were detected only in cells, 138 proteins were detected only in EVs, and 560 proteins were expressed in both cells and EVs (Fig. 4a). We speculate that 138 proteins only expressed in Homer1a^+^ EVs may play an important role in the function of Homer1a^+^ EVs. KEGG analysis of the 138 differentially expressed proteins revealed possible association with RAGE receptor binding and IL-17 signaling pathways (Fig. 4b). Ligand binding activates full-length RAGE and initiates downstream signal transduction pathways including NF-κB activation, thereby producing proinflammatory cytokines and inflammation [24]. Results of WB suggested that the expression levels of RAGE/NF-κB pathway-related proteins including RAGE, DIAPH1, P-IκBα^Ser32^, P-IκBα^Ser36^ and P-NF-κB^Ser536^ were higher in ICH mice compared with sham group, while Homer1a^+^ EVs treatment could reduce their expression levels (Fig. 4c). The same results were obtained in an in vitro ICH model in primary neurons (Fig. 4d). The transcription factor NF-κB is responsible for regulating transcription of IL-17A [25, 26]. Therefore, we examined the expression of IL17A in each group using ELISA. In peri-hemorrhagic tissues, it was found that ICH led to an increase in IL-17A expression, which was decreased by Homer1a^+^ EVs treatment (Fig. 4e). In the in vitro ICH model, although there was no significant difference in IL-17A expression levels between the supernatants of the 100 μg Homer1a^+^ EVs and ICH groups, IL-17A expression in the 200 μg Homer1a^+^ EVs group was significantly lower than that in the ICH group (Fig. 4f). IL-17A acts primarily by binding IL-17AR, one of the receptor subunits of IL-17A [27]. IL-17 binding induce the production of cytokines, chemokines, and other proteins by activating the NF-κB pathways [28–30]. We found that both IL-17AR and RAGE expression increased markedly after ICH and were spatially overlapping (Fig. 4g). However, EVs treatment significantly decreased the number of IL-17AR^+^ (Fig. 4h) and RAGE^+^ (Fig. 4i) cells in bleeding tissue compared with ICH group. The above results indicate that Homer1a^+^ EVs may improve the prognosis of ICH by inhibiting the RAGE/NF-κB/IL-17 signaling pathway in neurons.

### Homer1a^+^ EVs inhibit activation and nuclear translocation of NF-κB, thereby regulating transcription of IL-17A

**Fig. 5:**
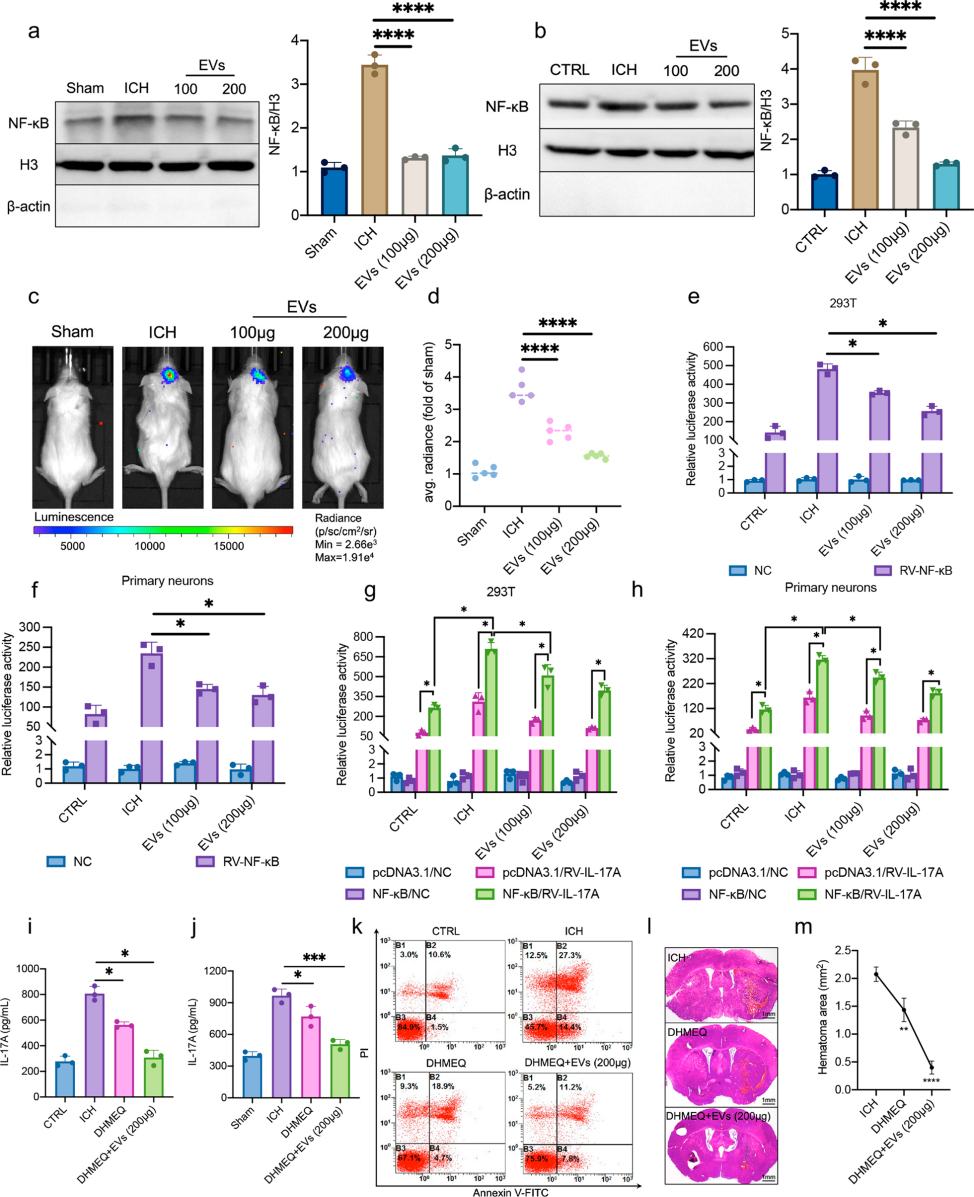
Homer1a^+^ EVs regulated activation and nuclear translocation of NF-κB. **a** The expression level and quantification of NF-κB in nucleus and cytoplasm (in vivo) was detected by WB [F (3, 8) = 164.3, P < 0.0001]. **b** The expression level and quantification of NF-κB in nucleus and cytoplasm (in vitro) was detected by WB [F (3, 8) = 122.60, P < 0.0001].** c** NF-κB-Luciferase reporter gene mice were used to detect the activation level of NF-κB.** d** Quantification of luciferase fluorescence intensity in panel c [F (3, 16) = 92.51, P < 0.0001]. **e** The regulation of ICH and Homer1a^+^ EVs on NF-κB activation was detected by double luciferase report assay in 293 T cells [F_interaction_ (3, 16) = 98.21, P < 0.0001]. **f** The regulation of ICH and Homer1a^+^ EVs on NF-κB activation was detected by double luciferase report assay in primary neurons [F_interaction_ (3, 16) = 25.92, P < 0.0001].** g** Regulation of IL-17A by NF-κB in different treatment groups in 293 T cells [F_interaction_ (9, 32) = 24.05, P < 0.0001].** h** Regulation of IL-17A by NF-κB in different treatment groups in primary neurons [F_interaction_ (9, 32) = 40.88, P < 0.0001].** i** Detection of IL-17A level in vitro by ELSIA [F (3, 8) = 85.94, P < 0.0001].** j** Detection of IL-17A level in vivo by ELSIA [F (3, 8) = 47.3, P < 0.0001].** k** Apoptosis level of primary inflammatory neurons after treatment with DHMEQ (5 μg/mL for 48 h) and Homer1a^+^ EVs.** l** HE staining of brain tissue of mice treated with DHMEQ (4 mg/kg) and Homer1a^+^ EVs.** m** Quantification of hematoma area intensity in panel **l** [F (2, 6) = 86.17, P < 0.0001]. The data were analyzed using one-way (**a**, **b**, **d**,** i**, **j** and **m**) or two-way (**e**, **f**, **g** and **h**) analysis of variance and all data are expressed as the mean ± standard deviation. *P < 0.05, **P < 0.01 ***P < 0.001 and ****P < 0.0001 represents a statistically significant difference between the two groups. ns: no statistical difference. The blots are representative of other replicates in those groups

NF-κB is activated and translocated to the nucleus where it acts [31–33]. Nuclear and Cytoplasmic Protein Extraction Kit was used to separate cytoplasm and nucleus. In both in vivo ICH models (Fig. 5a) and in vitro neuronal cultures (Fig. 5b), we found that NF-κB expression in the nucleus was significantly reduced after Homer1a^+^ EVs treatment compared to ICH group. To further assess the quantitative pattern of NF-κB activation, Transgenic NF-κB-Luciferase reporter gene mice were included. Due to the NF-κB-driven expression of the luciferase, the luminescence intensity directly correlates with NF-κB activity. As shown in Fig. 5c, compared to sham group, a significant increase of luciferase activity was observed in ICH mice on the third postoperative day. Homer1a^+^ EVs treatment for three consecutive days significantly reduced the fluorescence intensity of luciferase (Fig. 5d). In vitro 293 T (Fig. 5e) and primary neurons (Fig. 5f), the double luciferase report assay also showed that Homer1a^+^ EVs treatment inhibited the activation of NF-κB promoter induced by ICH.

In addition, an IL-17A-dependent transcriptional reporter containing five copies of an IL-17A response element located upstream of luciferase was used to investigate whether Homer1a^+^ EVs regulate transcription of IL-17A via NF-κB. It is found that in vitro 293 T (Fig. 5g) and primary neurons (Fig. 5h), overexpression of NF-κB promoted the expression of luciferase in IL-17A reporter plasmid, but the fluorescence intensity was inhibited by Homer1a^+^ EVs treatment. DHMEQ is a potent, selective and irreversible NF-κB inhibitor that binds covalently to cysteine residues. DHMEQ inhibited nuclear translocation of NF-κB and showed anti-inflammatory and anti-cancer activities [34]. Primary neurons treated with 10 μg/mL DHMEQ significantly decreased IL-17A content in cell supernatants, and the combination of DHMEQ and 200 ug Homer1a^+^ EVs resulted in lower expression of IL-17A compared with the ICH group (Fig. 5i). In vivo, three consecutive injections of DHMEQ or DHMEQ + Homer1a^+^ EVs after ICH in mice yielded similar results as in vitro (Fig. 5j). In addition, flow cytometry results indicated that DHMEQ or DHMEQ + Homer1a^+^ EVs treatment reduced ICH-induced apoptosis of primary neurons in vitro (Fig. 5k). The area of cerebral hemorrhage was declined after DHMEQ or DHMEQ + Homer1a^+^EVs treatment in vivo compared with ICH group and the difference is statistically significant (Fig. 5l, m). These results reveal that Homer1a^+^ EVs inhibit activation and nuclear translocation of NF-κB, thereby suppressing transcription of IL-17A.

### Homer1a^+^ EVs inhibit the binding ability of IL-17A: IL17-AR and RAGE: DIAPH1

Diaphanous related formin 1 (Diaphanous-1, DIAPH1) is one of the large actin nucleating/polymerizing protein family with multiple domains, which is characterized by catalytic FH2 domain. It is reported that the interaction of the RAGE cytoplasmic domain with DIAPH1 reduced diabetic complications in mice [35]. In addition, IL-17A acts primarily by binding IL-17AR and IL-17 binding induce the production of cytokines and chemokines by activating the NF-κB pathways [28–30]. Co-IP was performed to detect the binding ability of IL-17A:IL17-AR and RAGE:DIAPH1. Because the results above-motioned (Fig. 4c–i) suggested that there were differences in the expression of RAGE, DIAPH1, IL-17A and IL-17AR between the different treatment groups, sample loading between groups was adjusted to ensure that the protein levels of RAGE, DIAPH1, IL-17A and IL-17AR in the corresponding groups were consistent to ensure comparability of the results.

The results of Co-IP revealed that the binding ability of IL-17A: IL-17AR (Additional file 1: Fig. S2a-b) and RAGE: DIAPH1 (Additional file 1: Fig. S2c-d) increased in mice and primary neuronal ICH models. Interestingly, the binding ability of IL-17A: IL-17AR and RAGE: DIAPH1 in Homer1a^+^ EVs treatment group was inferior versus ICH group. The suppressive effect of Homer1a^+^ EVs on the protein was positively related to the dose. These data indicate Homer1a^+^ EVs inhibit the binding ability of IL-17A: IL17-AR and RAGE: DIAPH1, thereby inhibiting the inflammatory response induced by ICH.

### Homer1a^+^ EVs protect against the pathology, behavior, and survival rate in ICH Nestin^Cre^RAGE^fl/fl^ mice

To verify the role of RAGE in neurons, Nestin^Cre^RAGE^fl/fl^ transgenic mice were generated (Additional file 1: Fig. S3a). The ICH model was constructed and Homer1a^+^ EVs were treated according to the methods described previously (Additional file 1: Fig. S3b). We found that 200 μg Homer1a^+^ EVs treatment inhibited the expression of TNF-α (Additional file 1: Fig. S3c) and IL-1β (Additional file 1: Fig. S3d) in the brain tissue of ICH mice, while there was no significant difference between 100 μg Homer1a^+^ EVs treatment group and ICH group in the expression of IL-1β and TNF-α. Interestingly, the treatment of Homer1a^+^ EVs did not show superiority in the behavior (Additional file 1: Fig. S3e), survival rate (Additional file 1: Fig. S3f) of Nestin^Cre^RAGE^fl/fl^ mice and hematoma area of brain tissue (Additional file 1: Fig. S3g-h). The results of Nissl staining (Additional file 1: Fig. S3i-j) and TUNEL staining (Additional file 1: Fig. S3k-l) indicated that compared with ICH group, the apoptosis of ICH mice was effectively improved after 200 μg Homer1a^+^ EVs treatment, while there was no significant difference between 100 μg Homer1a^+^ EVs group and ICH group. Moreover, the treatment of Homer1a^+^ EVs did not show superiority in apoptosis of primary neurons (Additional file 1: Fig. S3m). These results indicated that Homer1a^+^ EVs may play a therapeutic role in ICH through RAGE pathway, and the deletion of RAGE in neurons weaken the therapeutic effect of Homer1a^+^ EVs.

### Assessment of inflammation and toxicity in Homer1a^+^ EVs treated mice

**Fig. 6:**
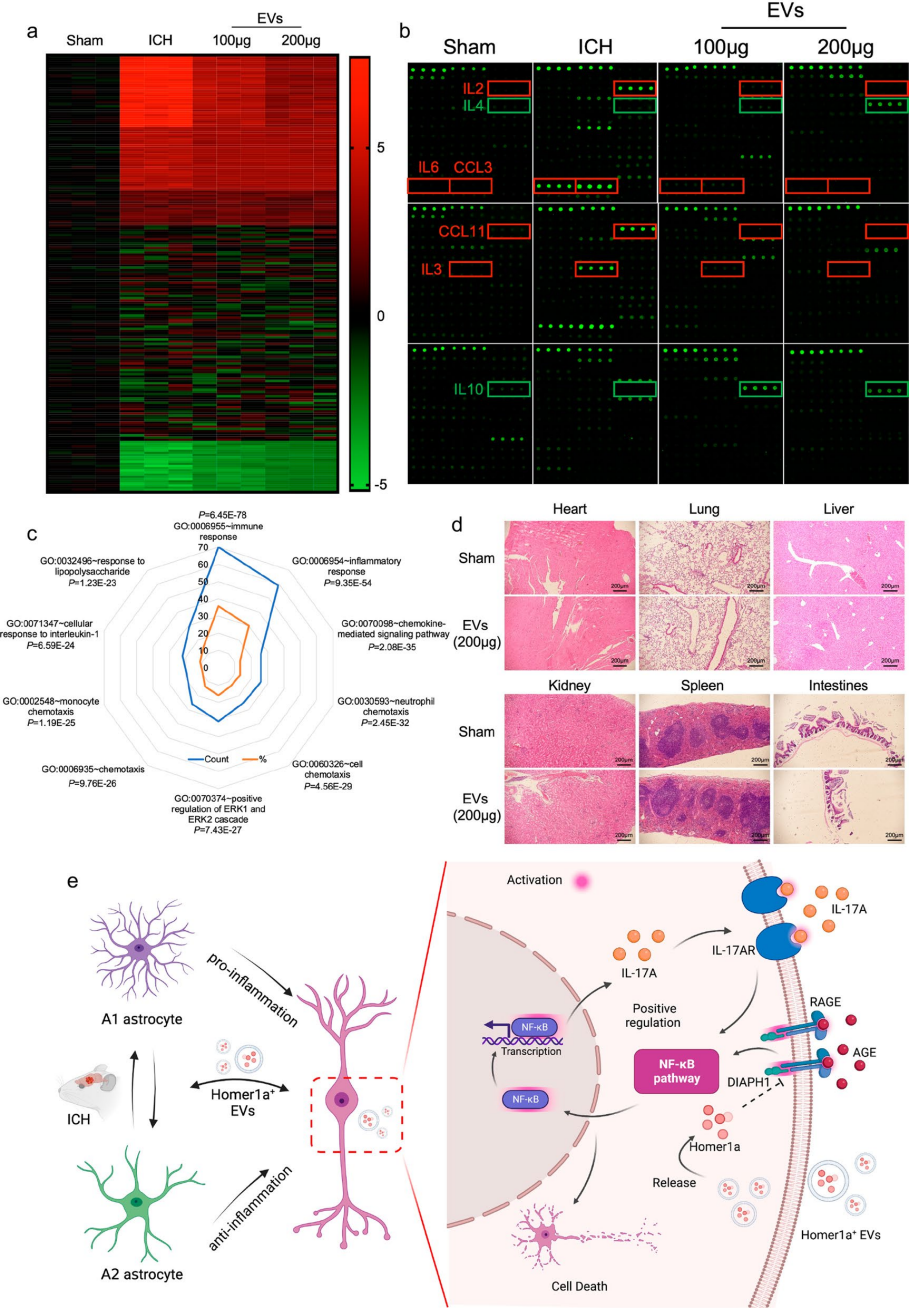
Inflammation and toxicity in Homer1a^+^ EVs treated mice.** a** Heat map of inflammatory factor microarray detection results including two hundred inflammatory indicators. **b** Fluorescent blot plots of representative inflammatory factors in each group. **c** GO analysis of differentially expressed genes. **d** HE staining of major organs, including the kidney, liver, lung, spleen, heart, and intestines in sham and EVs (200 μg) group. **e** Molecular mechanism diagram.

The brain tissues of wild type genotype ICH mice and mice treated with Homer1a^+^ EVs were used for inflammatory factor microarray detection to explore the effect of Homer1a^+^ EVs on inflammatory indicators in ICH. Two hundred inflammatory indicators were detected by using the mouse cytokine array Q4000. The microarray results showed that 79 pro-inflammatory factors increased, and 22 anti-inflammatory factors decreased in the brain tissue of mice after ICH (Fig. 6a). Compared with ICH group, the expression of 33 pro-inflammatory factors decreased in brain tissue of mice treated with Homer1a^+^ EVs including IL-2, IL-6, CCL3, CCL11 and IL-3. Interestingly, Homer1a^+^ EVs treatment promoted the expression of 22 anti-inflammatory factors including IL-4 and IL-10 (Fig. 6b). GO analysis was carried out on the differentially expressed genes between groups. The results showed that the differentially expressed genes were mainly involved in immune (6.45074E-78) and inflammatory reactions (9.35635E-54) according to the order of P value (Fig. 6c). In addition, we next examined the toxicity of Homer1a^+^ EVs on the major organs, including the kidney, liver, lung, spleen, heart, and intestines. The histological analysis of HE staining showed no evidence of toxicity of Homer1a^+^ EVs in vivo (Fig. 6d). The above experiments indicated that Homer1a^+^ EVs may participate in the post ICH treatment of mice by regulating anti-inflammatory and proinflammatory responses.

## Discussion

We first induced the transformation of astrocyte phenotype from A1 to A2 in vitro, and then Homer1a-pWPI plasmid was constructed and transfected into A2 astrocyte. Finally, EVs rich in Homer1a protein was successfully obtained, and the EVs have ideal stability and safety. The treatment of Homer1a^+^EVs at the bleeding site of ICH can effectively improve the inflammatory index, pathological characteristics, and survival time of mice after ICH. Homer1a^+^ EVs promotes the conversion of A1 astrocytes to A2 astrocytes in ICH mice. Homer1a^+^ EVs inhibits RAGE/NF-κB/IL-17 signaling pathway after ICH by suppressing nuclear translocation of NF-κB and the binding ability of IL-17A: IL17-AR and RAGE: DIAPH1 (Fig. 6e). Our results strongly support the use of EVs derived from A2 astrocytes as the versatile delivery system of Homer1a protein for the treatment of ICH.

A1/A2 astrocytes have mechanical support, barrier, immune response, and homeostasis maintenance [36]. The dual role of reactive astrocytes in disorders of central nervous system is well explained by A1/A2 phenotype astrocytes [18, 37]. A1 reactive astrocytes have the characteristics of inhibiting the formation of synapses, decreasing the phagocytic function, and secreting neurotoxins to induce the rapid neuronal death and oligodendrocytes [18, 38]. The neurotoxicity of type A1 reactive astrocytes has been widely found in neurodegenerative diseases such as Alzheimer’s disease [39] and Parkinson’s disease [40]. On the contrary, A2 astrocytes have neuroprotective effect [18, 41]. The gene expression of many neurotrophic factors in A2 type reactive astrocytes is up regulated, such as TGM1, CLCF1, S100A10, PTX3, CD109 and SPHK1, to promote the survival, growth, and differentiation of neurons [18, 42]. In vitro, neuroinflammation produced by systemic injection of lipopolysaccharide has been found to induce type A1 reactive astrocytes, whereas cerebral ischemia following middle cerebral artery occlusion induces type A2 reactive astrocytes in vivo [43]. However, unfortunately, A2 astrocyte model has not been successfully and effectively induced in vitro. Our study successfully induced A2 astrocyte model in vitro using hypoxia and co-culture with primary neurons, which provides a model basis for further study on the effect of A1/A2 astrocyte activation status on central nervous system diseases.

In recent years, with the deepening of exosomes research, it has been found that in addition to exosomes, micro vesicles, and apoptotic bodies are also involved in the transmission of intercellular information [44, 45]. Because of the overlap of the physical and chemical properties among the above three, more and more researchers put them into one category—EVs. It is reported that EVs/exosomes have therapeutic effects on ICH, and these EVs are derived from bone marrow mesenchymal stem cell (BMSC) [46, 47]. Ding et al. have found that EVs derived from BMSC alleviate neuroinflammation after diabetic intracerebral hemorrhage via the miR-183-5p/PDCD4/NLRP3 pathway [47]. Although stemness cell derived EVs may have better efficacy, uncertainty about differentiation raises long-term safety concerns. While our extracted EVs were derived from A2 astrocytes induced in vitro, and compared with that of BMSC, astrocyte differentiation was more stable. In addition, the physiological functions of A1/A2 astrocytes are completely different, and it is believed that A2 astrocyte derived EVs may have a better treatment for intracerebral hemorrhage compared with that of A1 astrocytes. Most importantly, we modified A2 astrocytes with Homer1a-pWPI overexpression plasmid using genetic engineering techniques. The protein level of Homer1a was higher in modified EVs than that in common EVs. In addition, Homer1a^+^ EVs were injected directly into the ICH site in the treatment phase. Compared to intravenous injection, orthotopic injection of EVs does not require consideration of additional distribution and consumption.

To verify the efficacy of Homer1a^+^ EVs in ICH mice and exclude the effect of Homer1a secretion by astrocytes themselves in mice, we generated astrocyte conditional knockout Homer1a mice (GFAP^Cre^Homer1^fl/−^Homer1a^±^). Up-regulation of Homer1a protein expression can cause the redistribution of Homer1b/c in synapses [8]. Because Homer1a and Homer1b/c share 186 amino acids in the gene sequence, it is our greatest difficulty to knock out Homer1a without affecting Homer1b/c when constructing transgenic mice. However, we made it. According to the method described in the study, [48, 49] exon 5 and intron 5 of Homer1-203 transcript are selected as editing regions. The exon 5-exon 9 cDNA sequence in Rat Homer1-204 transcript is knocked into the 3’ end of exon 5 using embryonic stem cell targeting strategy to selectively knock out Homer1a.

In recent years, more and more studies have shown that the interaction of various cells in the brain is very important for the physiological and pathological processes, especially the “cross-talk” between glial cells plays a central role in regulating inflammation in the brain [36]. For instance, in vitro studies have confirmed that microglia release TNF-α by paracrine means and induce the proliferation of surrounding astrocytes, and astrocyte culture medium can significantly reduce the response of microglia to oxidative stress [18, 50]. This regulatory pathway acts as a negative feedback mode of regulation, inhibiting microglial production of excessive oxidative free radicals, thereby reducing non-specific damage to neurons. In addition, Quintas et al. showed that when central nervous inflammation occurs, uracil nucleotides are released into the extracellular matrix, activating glial cell pyrimidine receptors, and promoting the development of their reactive phenotype, indicating that microglia-astrocytes affect chronic neuroinflammation and reactive gliosis through the pyrimidine receptor pathway [51]. In conclusion, both microglia and astrocytes have dual regulatory roles in neuroinflammatory responses. Activated microglia can either promote astrocyte activation or inhibit astrocyte activation. Similarly, activated astrocytes can either promote microglial activation or inhibit microglial activity. In our study, we observed that Homer1a^+^ EVs could promote the conversion of astrocyte phenotype from A1 to A2 in vivo and ameliorate neuronal apoptosis, but it is unknown whether Homer1a^+^ EVs exert effects on microglia in vivo. Although we found a significant therapeutic efficacy of Homer1a^+^ EVs on ICH, further cell-to-cell regulatory mechanisms remain to be investigated.

## Conclusions

Together, we constructed a platform for targeted delivery of Homer1a by using EVs and highlighted the potential of Homer1a^+^ EVs as a promising nanotherapeutic for ICH treatment. It was demonstrated that EV-medicated delivery exhibits unique advantages including improving the pharmacokinetic properties of Homer1a, making it more stable, safe, and targetable to injury sites, and extending the therapeutic targets of Homer1a^+^ EVs to include not only astrocytes but also neurons. Mechanistically, it was showed that Homer1a^+^ EVs promoted the conversion of A1 astrocytes to A2 astrocytes in ICH mice, inhibited the RAGE/NF-κB/IL-17 signaling pathway and consequently ameliorated the pathology, behavior, and survival rate in ICH. In addition, Homer1a + EVs also inhibited the binding ability of IL-17A: IL17-AR and RAGE: DIAPH1. Our study provides a novel insight and potential for the clinical translation of Homer1a^+^ EVs in the treatment of ICH or even for other neurological disorders.

### Supplementary Information

**Additional file 1. **This file includes: Figures S1 to S3 and additional "Materials and Methods" section.

## Data Availability

The datasets used and/or analyzed during the current study are available from the corresponding author on reasonable request.

## Abbreviations

ICH: Intracerebral hemorrhage
CNS: Central nervous system
EVs: Extracellular vehicles
DMEM: Dulbecco’s modified Eagle’s medium
NSE: Neuron-specific enolase
FBS: Fetal bovine serum
GFAP: Glial fibrillary acidic protein
ACM: Astrocyte conditional medium
NTA: Nanoparticle tracking analysis
TEM: Transmission electron microscopy
DHMEQ: Dehydroxymethylepoxyquinomicin
ELS: Erythrocyte lysis stimulation
PCR: Polymerase chain reaction
HE: Hematoxylin–eosin
WB: Western blot
qPCR: Quantitative polymerase chain reaction
Co-IP: Co-immunoprecipitation
ELISA: Enzyme linked immunosorbent assay
BMSC: Bone marrow mesenchymal stem cell
