# Supplementary material for: Extracellular vesicle encapsulated Homer1a as novel nanotherapeutics against intracerebral hemorrhage in a mouse model
Source: J Neuroinflammation. 2024 Apr 6;21:85. doi: 10.1186/s12974-024-03088-6 (PMC10999083; doi:10.1186/s12974-024-03088-6)
Supplement: Supplementary file 1 — Additional file 1. This file includes: Figures S1 to S3 and additional "Materials and Methods" section. [file 12974_2024_3088_MOESM1_ESM.docx]

**This PDF file includes:**

Figures. S1 to S3

Materials and Methods

**
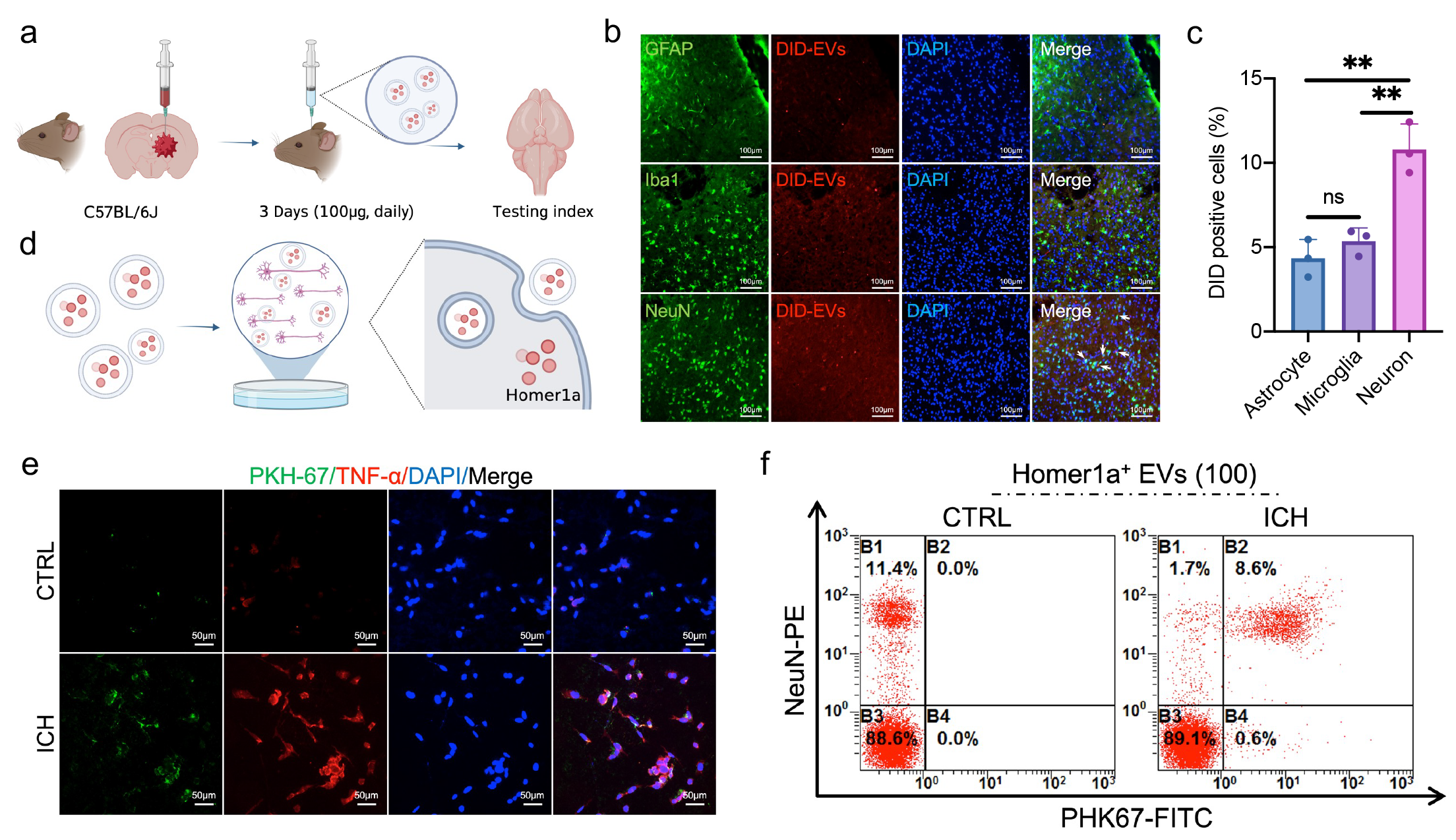
**

**Fig. S1 Homer1a+EVs are located in neurons.**

**a** Schematic diagram of C57BL/6J mice injected with Homer1a+ EVs. **b** Representative confocal images of the co-localization of Homer1a+ EVs with neurons (NeuN), microglia (Iba1), and astrocytes (GFAP). **c** Quantification of result in panel b [F (2, 6) = 26.10, P = 0.0011]. **d** Schematic diagram of co-culture of primary neurons and EVs. **e** Representative confocal images of PKH67-labeled Homer1a+ EVs in primary neurons inflammation model after 48 hours of incubation. **f** PKH67-labeled Homer1a+ EVs in primary neurons inflammation model after 48 hours of incubation. The data were analyzed using one-way analysis of variance and all data are expressed as the mean ± standard deviation. **P < 0.01 represents a statistically significant difference between the two groups. ns: no statistical difference.

**
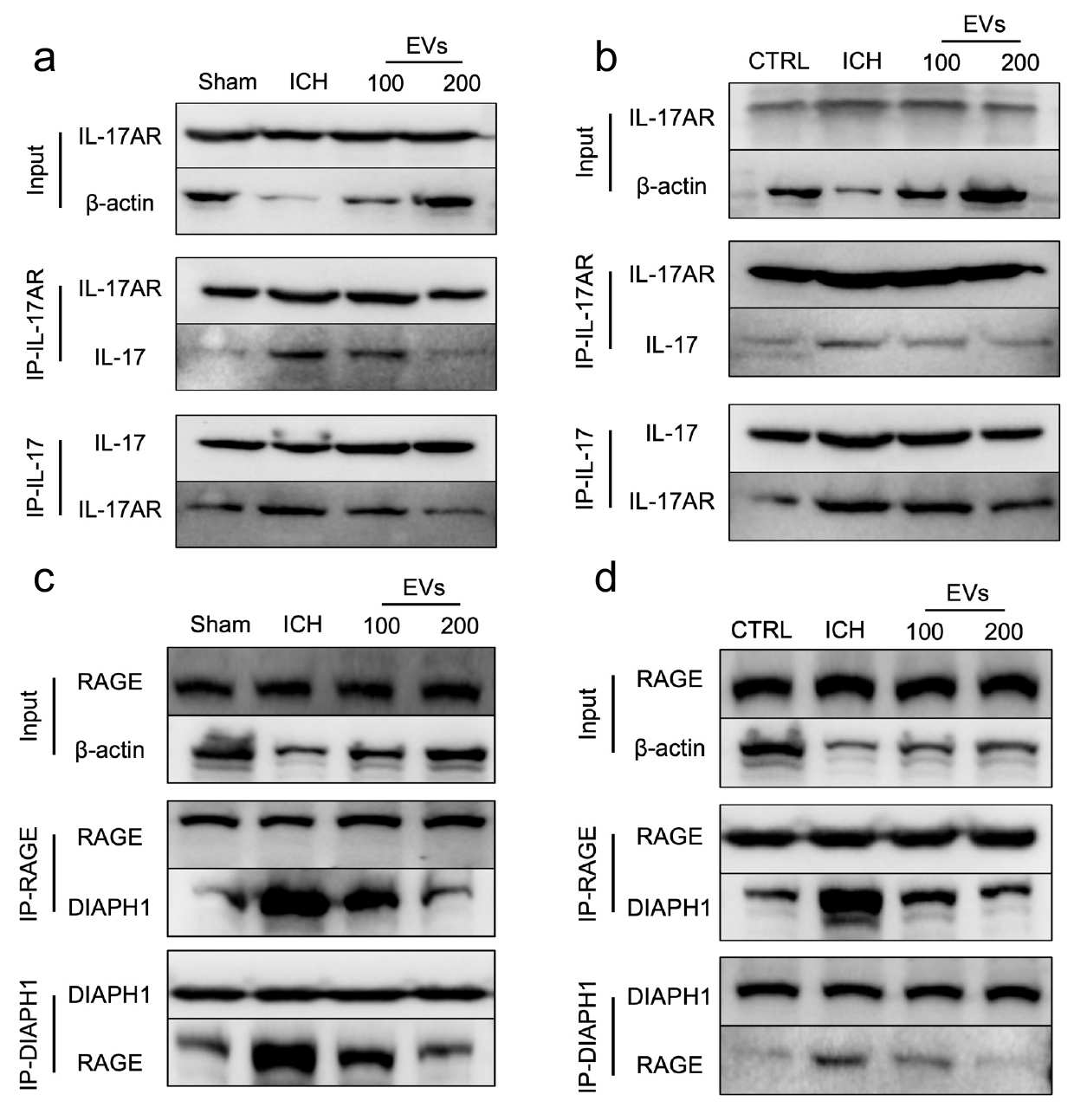
**

**Fig. S2 the binding ability of IL-17A: IL17-AR and RAGE: DIAPH1 were inhibited by Homer1a+ EVs.**

**a** Co-IP assay was used to detect the binding capacity of IL-17A and IL-17AR in vivo. **b** Co-IP assay was used to detect the binding capacity of IL-17A and IL-17AR in vitro. **c** Co-IP assay was used to detect the binding capacity of RAGE and DIAPH1 in vivo. **d** Co-IP assay was used to detect the binding capacity of RAGE and DIAPH1 in vitro. The blots are representative of other replicates in those groups.

**
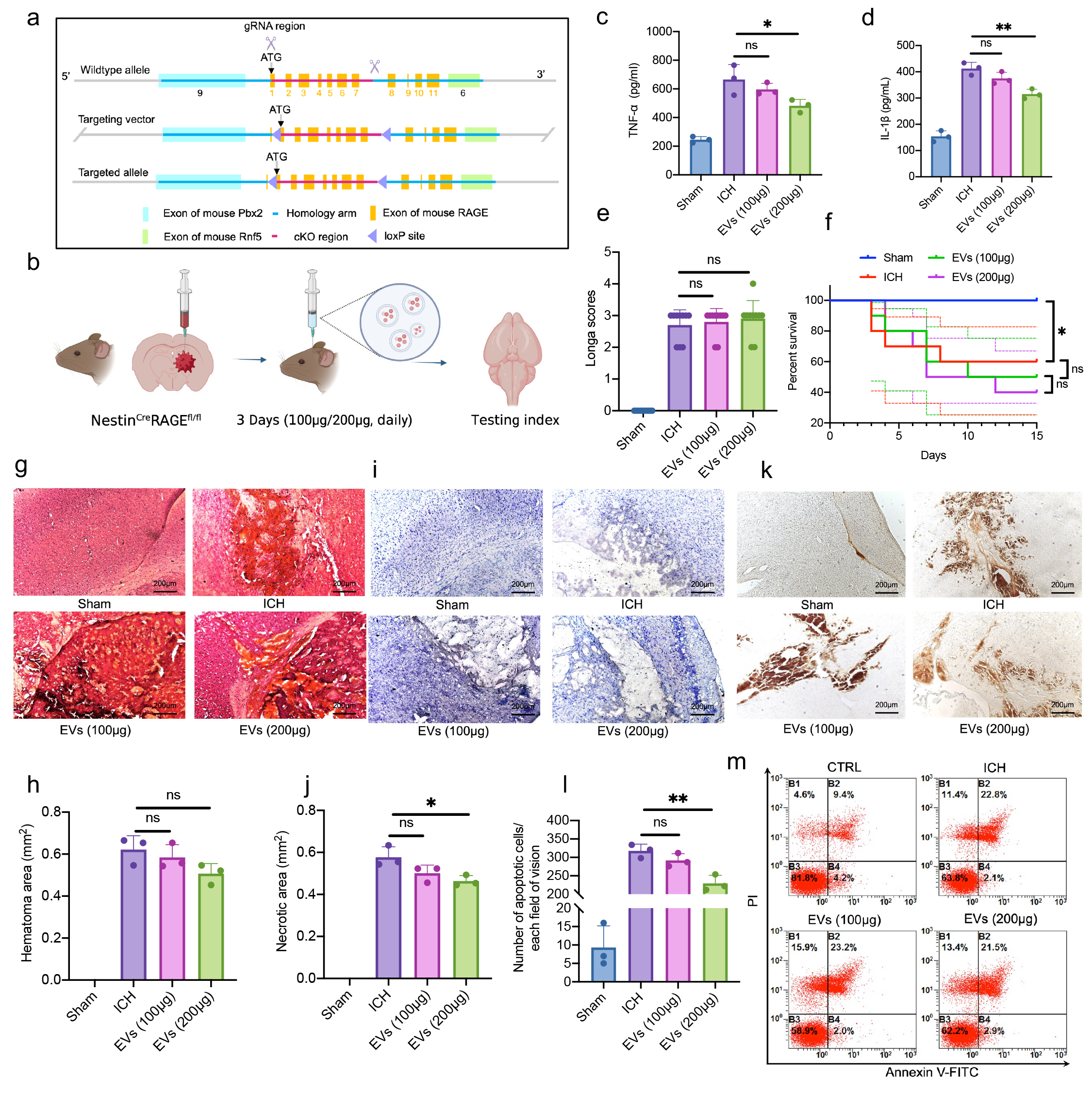
**

**Fig. S3 Homer1a^+^ EVs has therapeutic effect on ICH Nestin^Cre^RAGE^fl/fl^ mice.**

**a** Schematic diagram of RAGE conditional knockout Mouse model (C57BL/6J) by CRISPR/Cas-mediated genome engineering. **b** Schematic diagram of Nestin^Cre^RAGE^fl/fl^ mice injected with Homer1a^+^ EVs. **c** Detection of TNF-α level in brain tissue of each group by ELSIA [F (3, 8) = 26.69, P = 0.0002]. **d** Detection of IL-1β level in brain tissue of each group by ELSIA [F (3, 8) = 81.76, P<0.0001]. **e** Longa scores of mice in different groups [F (3, 36) = 107.3, P<0.0001]. **f** Survival curve of mice in each group (n = 10/group) [log-rank (Mantel–Cox) test: Chi-square = 7.424; df = 3; P = 0.0595]. **g** Representative photographs of HE staining of brain tissue in each group. **h** Quantification of result in panel g [F (3, 8) = 97.69, P<0.0001]. **i** Representative photographs of Nissl staining of brain tissue in each group. **j** Quantification of result in panel i [F (3, 8) = 178.4, P<0.0001]. **k** Representative photographs of TUNEL staining of brain tissue in each group. **l** Quantification of result in panel k [F (3, 8) = 198.2, P<0.0001]. **m** Representative photographs of flow cytometry of primary neuron in each group. The data were analyzed using one-way analysis of variance and all data are expressed as the mean ± standard deviation. *P < 0.05 and **P < 0.01 represents a statistically significant difference between the two groups. ns: no statistical difference.

**Supplemental material and methods**

**Identification of transgenic mice**

Tails of transgenic mice were cut and digested with proteinase K for 20 min at 55 °C, and further inactivated with protein K for 5 min at 100 °C. Polymerase chain reaction (PCR) was performed according to the protocol in One Step Mouse Genotyping Kit (Vazyme; China). The primer sequence information is as follows: Homer1 Flox (wild type: 232 bp; mutant: 300 bp): Primer-F: 5’-TGA GCT GGA CAC CCC CTG CC-3’, Primer-R: 5’- TGT TAA AAC AAT TAC ACC CGA TTC TT -3’; Homer1a-KO (Wild type: 485bp; mutant: 318bp): Primer1 (Wild type): 5’-TTG CTA AAG CGC TAC ATA GGA-3’, Primer2 (Common): 5’-GCC TTA TTG TGG AAG GAC TG-3’, Primer3 (Mutant): 5’-CCT TCC TGA AGC AGT AGA GCA-3’. RAGE Flox (wild type: 167 bp; mutant: 235 bp): Primers-F: 5’-CTC TGA CAG AAC GAG ATG GGA GAT-3’, Primers-R: 5’-CCA TAG AGC AAG AAC CAG CAC C-3’. GFAP-Cre (Wildtype: N.A. Targeted: 700 bp): Primer-F: 5’-TAG CCC ACT CCT TCA TAA AGC CCT-3’, Primer-R: 5’-GCT AAG TGC CTT CTC TAC ACC-3’. Nestin-Cre (wild type: 246 bp; mutant: 150 bp): Primer-WT: 5’-TTG CTA AAG CGC TAC ATA GGA-3’, Primer-MT: 5’-CCT TCC TGA AGC AGT AGA GCA-3’, Primer 3 (common): 5’-GCC TTA TTG TGG AAG GAC TG-3’. PCR results were detected by agarose gel electrophoresis. Three replicate experiments were performed for each mouse tail. ­­­­

**Hematoxylin-eosin (HE) staining, Nissl staining and TUNEL assay**

All these experiments were performed as previously described.^[1-3]^ Briefly, the frozen sections of mouse brain tissue with the largest bleeding area on the coronal plane were used for HE, Nissl and TUNEL staining. Hematoxylin and eosin staining solutions (BASO, Guangdong) were used for HE staining. 1% Toluidine blue (Beyotime, Shanghai) was used for Nissl staining. TUNEL kit (Beyotime, Shanghai) was used for TUNEL staining. The experimental procedures were performed in strict accordance with the manufacturer’s instructions.

**Immunohistochemistry**

Immunohistochemical analysis was performed as previously described.^[1]^ Three mice of each group were anesthetized after different treatment and the frozen sections of mouse brain tissue with the largest bleeding area on the coronal plane were used for immunofluorescence analysis. The following antibodies were used: mouse anti-Iba1 (1:250; GeneTex), mouse anti-GFAP (1:300; CST), mouse anti-NeuN (1:300; CST), rabbit anti-S100A10 (1:300; Proteintech), rabbit anti-C3 (1:200; Abcam), rabbit anti-TNF-α (1:5000; Abcam), rabbit anti-IL-17AR (1:50; Abcam), mouse anti-RAGE (1:100; Millipore) and donkey anti-rabbit/mouse IgG (H + L) highly cross-adsorbed secondary antibody, Alexa Fluor Plus 488/555 (1:1000; Invitrogen).

**Western blot (WB)**

WB was performed as previously described.^[1]^ EVs, primary astrocytes, primary neurons and the brain tissue around the bleeding site in each group were harvested for protein extraction. The following antibodies were used: rabbit anti-Homer1a (1:1000; Thermo), rabbit anti-CD9 (1:1000; CST), rabbit anti-CD63 (1:1000; Abcam), rabbit anti-CD81(1:1000; CST), rabbit anti-TSG101(1:1000; CST), rabbit anti-C3 (1:1000; Abcam), rabbit anti-S100A10 (1:1000; Proteintech), rabbit anti-RAGE (1:1000; CST), rabbit anti-DIAPH1 (1:1000; Abcam), rabbit anti-P-IKBα^Ser32^ (1:1000; Abcam), rabbit anti- P-IKBα^Ser36^ (1:1000; Abcam), rabbit anti-IKBα (1:1000; Abcam), rabbit anti-P-NF-κB^Ser536^ (1:1000; CST), rabbit anti-NF-κB (1:1000; GeneTex), rabbit anti-H3 (1:1000; Abcam), rabbit anti-IL-17AR (1:1000; Abcam), rabbit anti-IL-17 (1:1000; Abcam), mouse anti-β-actin (1:10,000; Abcam) and goat anti-mouse/rabbit secondary anti- body (1:10,000; Abcam).

**Quantitative polymerase chain reaction (qPCR)**

Mice with different treatments were anesthetized at 3 days after ICH induction, and the brain tissue around the bleeding site was used for qPCR. Brain tissue and different modified primary astrocytes were harvested for RNA extraction using TRIzol (ambion, Thermo) reagent. Reverse transcription was performed according to the protocol of the HiScript II Q Select RT SuperMix for qPCR (+gDNA wiper) kit (Vazyme, China). qPCR was performed according to the protocol of the ChamQ SYBR Color qPCR Master Mix (Low ROX Premixed) kit (Vazyme, China). The primers for mRNA were as follows: C3-F: “AAC AAG CTC TGC CGT GAT GA”, C3-R: “GCC TGA CTT GAT GGT CTG CT”; S100A10-F: “TGA GAG TGC TCA TGG AAC GG”, S100A10-R: “AGA AAG CTC TGG AAG CCC AC”; β-actin-F: “AAC AGT CCG CCT AGA AGC AC”, β-actin-R: “CGT TGA CAT CCG TAA AGA CC”. The expression of related RNAs was calculated using the 2^−ΔΔCt^ method, and β-actin was used as a control.

**Longa scores**

Mice with different treatment were scored behaviorally at 3 days after ICH induction. The scores were calculated using the Longa method. inability to walk automatically, with loss of consciousness: 4 points; dumping to the paralyzed side during walking: 3 points; circling to the paralyzed side during walking: 2 points; inability to fully extend the forepaws on the paralyzed side: 1 point; No neurological deficit: 0 points.

**Co-immunoprecipitation (Co-IP)**

Co-IP was performed as previously described.^[1, 4]^ Briefly, an appropriate amount of brain tissue was taken from the bleeding site on the third day after different treatment and 3 ml pre-cooled RIPA lysate was added. The tissue was ground using a tissue homogenizer and placed on ice for 30 min. Tissue debris was removed by centrifugation at 10,000×g for 10 min in 4 °C. one ml supernatant was mixed with 0.2 μg primary antibody in 1.5 mL EP tube and incubated at 4 °C for 1 h. The solution was incubated overnight with 20 μL protein A/G Plus-agarose (Santa Cruz; China) on a rotary mixer at 4 °C. The beads were collected by centrifugation at 2500 rpm for 5 min in 4 °C and washed thrice with PBS. After washing the beads, 40 μL electrophoresis loading buffer was added and boiled in a 100 °C water bath for 10 min. After centrifugation at 2500 rpm for 5 min in 4 °C, the supernatant was collected and used for Western blot.

**Enzyme linked immunosorbent assay (ELISA)**

Cell supernatants from the different treatment groups were harvested for ELISA. Mice were anesthetized after different treatment and the brain tissue around the bleeding site was used for ELISA. ELISA was performed in strict accordance with the manufacturer’s instructions. The following ELISA kits were used for detection: Rabbit anti-Homer1a antibody (Synaptic Systems, Germany), Mouse IL-1 beta ELISA Kit (Abcam, UK), Mouse TNF alpha ELISA Kit (Abcam, UK) and Mouse IL-17A ELISA Kit (Abcam, UK).

**Flow cytometry**

Flow cytometry was used to detect the difference of apoptosis between different groups. Annexin V-FITC/PI Apoptosis Detection Kit was purchased from Vazyme (China, Nanjing). The experimental steps were carried out in strict accordance with the instructions.

Flow cytometry was used to detect the difference of protein expression between different groups. Cells after different treatments were collected and washed with PBS in Eppendorf (EP) tube. FIX & PERM™ Cell permeability kit (Thermo, USA) is used to immobilize cells and increase cell permeability. Cell Staining Buffer (BioLegend, USA) is used to prepare antibody. Cells with 1mL antibody working solution were put it in Rotation Mixer at 4 ℃ overnight. The cells were washed with PBS and then detected by flow cytometry. Rabbit anti-NeuN, Rabbit anti-C3, Mouse anti-S100A10, Goat anti-rabbit IgG (H&L) (PE) / (APC), Goat anti-mouse IgG (H&L) (FITC) were purchased from Abcam.

**Double luciferase reporter assay**

The NF-κB firefly-luciferase reporter vector (RV-NF-κB), NF-κB overexpression plasmid (NF-κB), The IL-17A firefly-luciferase reporter vector (RV-IL-17A), blank control vector pcDNA3.1 (pcDNA3.1) and Renilla reporter constructs were designed and purchased from Genechem Co., Ltd (China, Shanghai). Renilla activity was used to normalize luciferase reporter activity. The promoter-less firefly-luciferase vector pGL4.15 served as the negative control (NC). Two corresponding plasmids were co-transfected into modified primary neurons and 293T using jetPRIME transfection reagent (PolyPlus, France). After 48h, luciferase activities were examined using the Dual-Luciferase Reporter Assay Kit (Promega). Assays were performed on cells in three wells for each experiment to obtain an average count, and in three independent biological replicates.

**Shotgun experiments**

Shotgun experiments were performed with the assistance of GeneChem Co., Inc. (Shanghai, China). Briefly, the donor A2 astrocytes and EVs to be tested were subjected to mass spectrometry after chemical digestion. The raw data obtained was then imported into Proteome Discoverer 2.2 (Thermo Fisher Scientific) for protein identification, then embedded Mascot 2.6 engines was used for database searches (Proteome Discoverer Version 2.2. Thermo Fisher Scientific Inc. 2017). Protein identification was performed using reviewed database. The search parameters included trypsin as the enzyme used to generate peptides with a maximum of 2 missed cleavages permitted. A precursor mass tolerance of 10 ppm was specified and 0.05 Da tolerance for MS2 fragments. The carbamidomethyl (C) was set as a fixed modification and variable modifications were Oxidation(M) and Acetyl (Protein N-term). Protein was considered as positively identified if peptide score of specific peptides reached the significance threshold FDR = 0.01.

**Protein chip assay**

The brain tissue around the bleeding site was used for the detection of inflammatory factors and cytokines. The mouse cytokine array Q4000 was purchased from RayBiotech (QAM-CAA-400; USA). The experimental procedures were performed in strict accordance with the manufacturer’s instructions.

1. Fei, X., et al., *The role of Toll-like receptor 4 in apoptosis of brain tissue after induction of intracerebral hemorrhage.* Journal of neuroinflammation, 2019. **16**(1): p. 234.

2. Fei, X., et al., *Homer1 promotes the conversion of A1 astrocytes to A2 astrocytes and improves the recovery of transgenic mice after intracerebral hemorrhage.* Journal of Neuroinflammation, 2022. **19**(1): p. 1-18.

3. Fei, X., et al., *TLR4 Deletion Improves Cognitive Brain Function and Structure in Aged Mice.* Neuroscience, 2022. **492**: p. 1-17.

4. Fei, X., et al., *TRIM22 orchestrates the proliferation of GBMs and the benefits of TMZ by coordinating the modification and degradation of RIG-I.* Molecular Therapy - Oncolytics, 2022. **26**: p. 413-428.
